# Single‐Molecule Ex Situ Atomic Force Microscopy Allows Detection of Individual Antibody–Antigen Interactions on a Semiconductor Chip Surface

**DOI:** 10.1002/anbr.202000035

**Published:** 2020-12-18

**Authors:** Ming-Pei Lu, Ying-Ya Weng, Yuh-Shyong Yang

**Affiliations:** ^1^ Taiwan Semiconductor Research Institute National Applied Research Laboratories Hsinchu 30078 Taiwan; ^2^ Institute of Biomedical Engineering National Chiao Tung University Hsinchu 30010 Taiwan; ^3^ Department of Biological Science and Technology National Chiao Tung University Hsinchu 30010 Taiwan

**Keywords:** antibody–antigen interactions, ex situ atomic force microscopy, hepatitis B virus X protein, IgG antibody, semiconductor chips, single-molecule detections

## Abstract

Although in situ atomic force microscopy (AFM) allows single‐molecule detection of antibody–antigen binding, the practical applications of in situ AFM for disease diagnosis are greatly limited, due to its operational complexity and long operational times, including the execution time for the surface chemical/biological treatments in the equipped glass liquid cell. Herein, a method of graphically superimposed alignment that enables ex situ AFM analysis of an immobilized antibody at the same location on a semiconductor chip surface before and after incubation with its antigen is presented. All of the required chemical/biological treatments are executed feasibly using standard laboratory containers, allowing single‐molecule ex situ AFM detection to be conducted with great practicality, flexibility, and versatility. As an example, the analysis of hepatitis B virus X protein (HBx) and its IgG antibody is described. Using ex situ AFM, individual information on the topographical characteristics of the immobilized single and aggregated IgG antibodies on the chip surface is extracted and the data are analyzed statistically. Furthermore, in a statistical manner, the changes in AFM‐measured heights of the individual and aggregated IgG antibodies that occur as a result of changes in conformation upon formation of IgG–HBx complexes are investigated.

## Graphical abstract

An ex situ atomic force microscopy (AFM)–molecule chip technique integrated with a standard manufactured semiconductor chip and a graphically superimposed alignment technique allows the ex situ AFM analysis detection of antibody–antigen interactions at the single‐molecule level. This approach is significantly superior to conventional in situ AFM analysis detection in terms of its practicality, flexibility, and versatility.

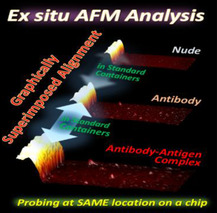

## 1 Introduction

Humans have always struggled with threats from infectious diseases. Thanks to scientific advances in biological and medical technologies, as well as global collaborations between nations, the effects of lethal infectious diseases seemed to be highly controllable. Suddenly, coronavirus disease 2019 (COVID‐19), caused by the severe acute respiratory syndrome coronavirus 2 (SARS‐CoV‐2), widely and rapidly spread over the world, seriously infecting more than ten million people and resulting in more than half a million people dying within the first six months of the pandemic.^[^ ^1^ ^]^ In addition to its effects on human health and life, COVID‐19 has seriously restricted global economic activity, due to a lack of vaccines and highly effective medicines. This event has highlighted our need to learn how to effectively control and suppress the spreading of future emerging pandemics in their early stages. Although changes to our daily habits have helped (in particular, social distancing, washing hands frequently, and wearing masks), we need to develop inexpensive, rapid, and ultra‐sensitive technologies for disease diagnosis to actively screen out infected persons, thereby, effectively monitor and block the early‐stage transmission of infectious diseases, and possibly even extinguish the infection entirely.

Many concepts for the next‐generation detection of infectious diseases have appeared over the decades.^[^ ^2^, ^3^, ^4^, ^5^, ^6^, ^7^, ^8^, ^9^ ^]^ In particular, surface analysis techniques based on atomic force microscopy (AFM) have been adopted widely for the detection of antibody–antigen interactions.^[^ ^10^, ^11^, ^12^, ^13^, ^14^, ^15^, ^16^ ^]^ These approaches have relied on 1) using a molecule‐coated AFM tip to probe the changes in the contacting force that occurs when molecules from the tip specifically bind to molecules on the surface^[^ ^3^, ^11^, ^12^ ^]^ and 2) characterizing variations in AFM topography mapping profiles that occur upon the conformational changes arising from specific binding between antigens and antibodies.^[^ ^14^, ^15^, ^16^ ^]^ For example, in situ AFM analysis has allowed such detection at the single‐molecule level. In this approach, AFM topographical analysis at the same location, before and after incubation with the antigen, can be used to identify variations in the topographical characterization of an individual antibody. To do so, the AFM system requires integration of a glass liquid cell to function as a solution transport system for the chemical/biological treatments required for detection.^[^ ^15^ ^]^ The analysis chip must be placed in firm contact with the glass liquid cell during the whole detection procedure to ensure that the same area is maintained for AFM analysis. The practical applications of in situ AFM for disease diagnosis are greatly limited, however, due to its operational complexity and long operational times, including the time wasted when executing the required chemical/biological treatments through the glass liquid cell system.

In this article, we present an ex situ AFM–molecule chip technique integrated with a standard manufactured semiconductor chip and a graphically superimposed alignment technique for the ex situ AFM analysis detection of antibody–antigen interactions at the single‐molecule level. This approach is superior to conventional in situ AFM analysis detection in terms of its practicality, flexibility, and versatility. The graphically superimposed alignment technique ensures that the ex situ AFM analysis occurs at the same location on a semiconductor chip surface before and after incubation with the antigen; therefore, all of the required chemical and biological treatments can be executed in standard laboratory containers—and not in the glass liquid cells required for in situ AFM analysis. A designed pattern on the chip surface functions as a graphically superimposed mark for precise alignment of the AFM mapping images recorded at each stage of the detection procedure, thereby enabling stepwise exploration of the evolution of the topographical characteristics at every location on the same AFM analysis area. To test the feasibility of this ex situ AFM–molecule chip technique at detecting the specific interactions between an antibody and a small‐sized protein at the single‐molecule level, we used the hepatitis B virus X protein (HBx) as our antigen. Experimental observations and statistical analysis revealed that an individual IgG antibody covalently immobilized on an aldehyde‐terminated chip surface, presumably in a predominantly head‐on orientation, and possessed an AFM height of 5.416 nm. Furthermore, we also used a statistical approach to obtain information about the change in the AFM height (an average increase of ≈1 nm) of an individual IgG antibody/aggregate that arose, presumably, from the conformational change that occurred upon the formation of the IgG–HBx complex. We believe that this ex situ AFM–molecule chip technique based on graphically superimposed alignment will offer opportunities for next‐generation single‐molecule disease diagnosis while also providing useful information regarding the behavior of individual antibodies and antigens from the single‐molecule perspective.

## 2 Results and Discussion

**Figure 1:**
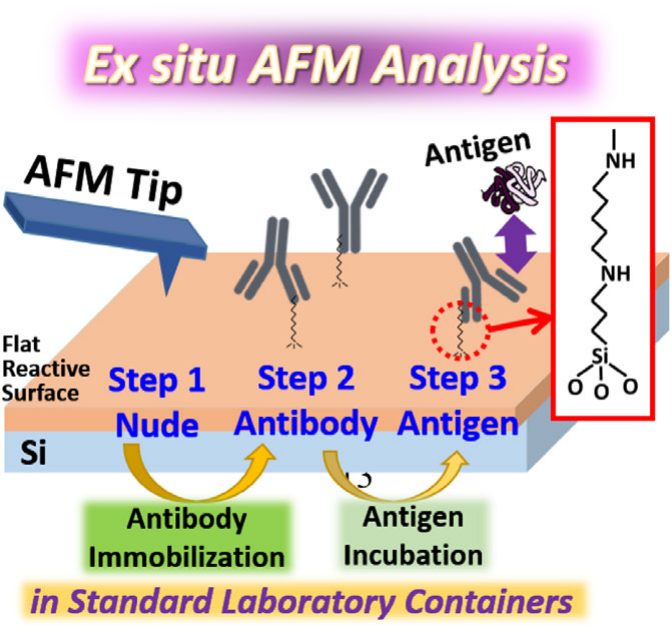
Schematic representation of the ex situ AFM–molecule chip technique. AFM analysis is conducted for the nude, antibody‐immobilized, and antigen‐incubated surfaces. The chemical structure of the APTES‐GA linker used to immobilize the antibodies is shown in the red box.

**Figure** **1** shows a conceptual depiction of our proposed experimental system for the ex situ AFM–molecule chip technique. In the first step of ex situ AFM analysis, the 3D topographical characteristics of the analysis area on the semiconductor chip are measured using AFM to confirm its roughness and cleanliness. Notably, the selected micrometer‐sized analysis area featuring the designed alignment pattern on the chip surface can be identified through optical microscopy. A flat reactive surface of SiO_2_ is preferred when attempting to identify a small molecule on a chip; such a surface can usually be obtained through nanoscale controllability of the thin‐film growth and etching processes when using standard semiconductor manufacture techniques. After the initial AFM analysis, chemical treatment processes for antibody immobilization on the SiO_2_ surface are conducted in standard laboratory containers. A second AFM analysis is then conducted to identify the immobilized antibody on the SiO_2_ surface, by means of variations in the AFM topographical mapping. Subsequently, the antigen is incubated of the chip for a period of time, followed by reloading of the chip onto the AFM sample stage for the third AFM analysis. From the viewpoint of applications in practical diagnosis, when compared with the typical in situ AFM analysis,^[^ ^15^, ^17^ ^]^ this ex situ AFM–molecule chip technique is more flexible in terms of implementing the chemical and biological treatments required for detection, while also requiring less total time for AFM usage for each detection, thereby enhancing the detection capacity.

**Figure 2:**
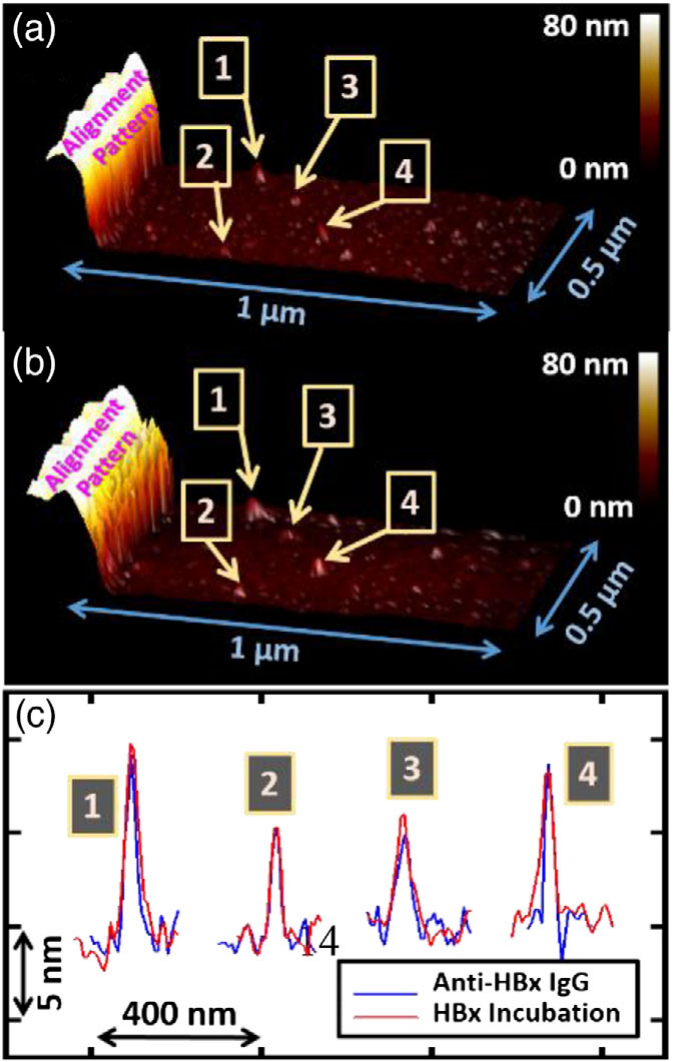
a,b) 3D AFM topographical mapping of the same analysis area, containing the designed alignment pattern, after (a) immobilization of anti‐HBx IgG and (b) incubation with the HBx protein. The four selected AFM peaks are denoted as peaks 1–4. c) Cross‐sectional AFM topographical profiles of peaks 1–4 recorded from the images in (a) and (b), denoted as blue and red lines, respectively. The *x*‐ and *y*‐axes represent the relative lateral and vertical displacements, respectively, of the AFM scans.

The main challenge when developing this ex situ detection procedure was to determine how to probe the topographical characteristics of the same analysis area on the chip at different stages. We investigated the concept of graphically superimposed alignment to ensure precise alignment of designed patterns in the ex situ AFM topographical mappings at each stage of the detection procedure. We wanted the alignment pattern on the chip surface to be readily identified by the optical microscopy component of the AFM system to facilitate the movement of the AFM tip to the analysis area. Furthermore, because the designed pattern was necessary for precisely aligning the ex situ AFM topographical mappings at each stage of detection, we wanted the pattern to be located within the AFM analysis area. Using this approach, we expected that any topographical differences at the same surface location could be identified between the various stages of the detection procedure. To test the feasibility of the ex situ AFM–molecule chip for single‐molecule disease diagnosis, we used HBx as a model small‐molecule antigen. The HBx protein plays an important role in hepatitis B virus (HBV) infection, one of the leading causes of hepatocellular carcinoma (HCC).^[^ ^18^, ^19^, ^20^ ^]^ Therefore, we suspected that our approach would also be helpful for understanding the interactions of the anti‐HBx IgG and HBx from the single‐molecule perspective. Experimental Section provides details of the chip fabrication, surface chemical modification, antibody immobilization, antigen incubation, and AFM analysis. Supporting Information provides a detailed description of the graphically superimposed alignment procedure of the ex situ AFM topographical mapping. **Figure** **2**a shows the 3D AFM topographical mapping of the analysis area of the chip after immobilization of the anti‐HBx IgG antibody. It is evident that several peaks appeared randomly on the SiO_2_ surface, as revealed in the AFM image of the analysis area (Figure 2a). Using the graphically superimposed alignment technique to align the AFM topographical mapping images to the same analysis area before and after immobilization of anti‐HBx IgG, we could reasonably attribute the peaks that appeared only after the anti‐HBx IgG immobilization in the AFM topographical mapping, as shown in Figure 2a, to immobilized IgG on the SiO_2_ surface. Furthermore, we conducted sequential chemical and biological procedures on the bare SiO_2_/Si chip and then analyzed the topographical features using AFM; the images of the nude, APTES‐terminated, APTES‐GA–terminated, and antibody‐immobilized surfaces confirmed that the peaks that were at least a few nanometers in height and tens of nanometers in width that appeared only after immobilization of the IgG antibody. Subsequently, through graphically superimposed alignment, we conducted AFM topographical mapping, over the same analysis area, of the antibody‐immobilized chip surface after incubation with the HBx antigen protein (Figure 2b). Figure 2c shows the cross‐sectional AFM topographical profiles obtained after scanning through the peak maxima of the four peaks in Figure 2a,b, plotted as blue and red lines, respectively. Interestingly, we could observe magnitudes of the AFM peak heights ranging from few to tens of nanometers through a single analysis on the same anti‐HBx immobilized chip. The topographical profile of peak 2 was approximately the same before and after incubation with HBx; thus, not all of the peak profiles were modified after incubation. These images confirm that the ex situ AFM–molecule chip technique can, indeed, be a precise and reliable experimental tool for probing changes in the topographical profiles of antibodies after incubation with their antigens. Notably, any interference arising from physical adsorption of the antigen (or other molecule) on the chip surface can be excluded when using the ex situ AFM–molecule chip technique because of the graphically superimposed alignment; thereby, we can focus directly on the AFM height variations of the immobilized antibodies on the analytical surface—an essential feature for the purpose of single‐molecule disease detection.

**Figure 3:**
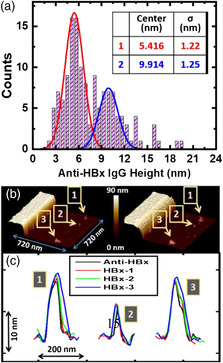
a) Histogram of the statistical distribution of AFM heights of the individual peaks in the 3D AFM topographical mappings of the IgG‐immobilized chip surface, obtained from 139 data points. The red and blue lines centered at 5.416 and 9.914 nm, fitted using Gaussian distributions, appear to correspond to single IgG antibodies and IgG aggregates of a few molecules, respectively. Inset table: Fitting parameters of the two main peaks. b) 3D AFM topographical mapping of the same area of the antibody‐immobilized surface prior to HBx incubation (left‐hand side) and after three successive HBx incubations (right‐hand side). The three selected AFM peaks are denoted as Nos. 1–3. c) Evolution of the cross‐sectional AFM peak profiles of the three selected species after antibody immobilization (black line) and one (red line), two (green line), and three (blue line) HBx incubations. The *x*‐ and *y*‐axes represent the relative lateral and vertical displacements, respectively, of the AFM scans.

To obtain greater molecular‐level insight into the AFM peaks that appeared in the 3D AFM topographical mapping, we examined the statistical distribution of the magnitudes of the individual peak heights measured from several chips. **Figure** **3**a shows two main peaks in the histogram. We used Gaussian distribution functions to fit those two experimental peak profiles as red (first peak) and blue (second peak) lines. The fitted parameters for the heights at the centers (and standard deviations, *σ*) for the first and second peaks were 5.416 nm (1.22 nm) and 9.914 nm (1.25 nm), respectively (inset table in Figure 3a). The peak center of 5.416 nm for the first peak is consistent with previously reported thicknesses of an IgG antibody monolayer immobilized on the aldehyde‐terminated substrate^[^ ^21^ ^]^ and heights of individual IgG antibodies extracted using AFM.^[^ ^16^, ^22^, ^23^, ^24^, ^25^, ^26^, ^27^, ^28^ ^]^ Thus, we attribute the first peak in the statistical distribution of the experimentally determined AFM peak heights to single anti‐HBx IgG antibodies immobilized on the chip surface. Furthermore, by comparing the cross‐sectional AFM profiles of various orientations of individual IgG antibodies on the chip surface,^[^ ^24^, ^25^, ^26^, ^27^, ^28^ ^]^ we suggest a predominantly head‐on orientation for the single immobilized IgG antibodies on our surface—consistent with previously reported observations of predominantly head‐on orientation for IgG antibodies immobilized on aldehyde‐terminated chip surfaces, resulting from the reactivity of the amino group at the *N*‐terminus of the IgG being higher than that of its lysine residues.^[^ ^26^, ^29^ ^]^ In addition, we attribute the second peak centered at 9.914 nm in Figure 3a to aggregates of a few IgG antibodies. Next, we designed a series of sequential HBx incubation experiments on the antibody‐immobilized chip to gain insight into the interactions of the anti‐HBx IgG aggregate with HBx protein. Figure 3b shows 3D AFM topographical mappings of the same area of the antibody‐immobilized chip surface before (left‐hand side) and after conducting three HBx incubations (right‐hand side). We selected a single IgG (No. 2) and two IgG aggregates (Nos. 1 and 3), as denoted in Figure 3b, for further investigation. Figure 3c shows the cross‐sectional AFM peak profiles of those three selected species after antibody immobilization (black line) and the first (red line), second (green line), and third (blue line) HBx incubations. The cross‐sectional topographical profile of the single IgG (No. 2) was modified only after the first incubation, whereas the cross‐section topographical profiles of the IgG aggregates (Nos. 1 and 3) increased after each incubation with HBx, consistent with more antigen binding sites present in an IgG aggregate than in a single IgG unit.

**Figure 4:**
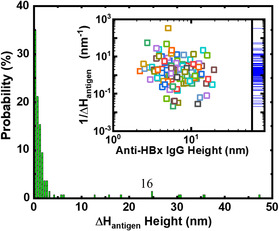
Histogram of the probability distribution of the change in amplitude (Δ*H*_HBx_) of the AFM‐measured anti‐HBx height after incubation with HBx. Inset: Plot of 1/Δ*H*_HBx_ with respect to the corresponding individual anti‐HBx AFM‐determined peak height, obtained from 137 data points; a rug plot of all of the data points for 1/Δ*H*_HBx_ is displayed along the *y*‐axis on the right‐hand side, revealing statistically that the maximum density distribution of the values of Δ*H*_HBx_ appeared at ≈1 nm.

**Figure** **4** shows the probability distribution of the changes in the amplitude of the AFM peak heights upon incubation with HBx (Δ*H*_HBx_). Most of the values of Δ*H*_HBx_ were less than 5 nm; we attributed the several data points having values of Δ*H*_HBx_ of a few tens of nanometers to the binding of the HBx aggregates to an individual IgG antibody/aggregate. The inset in Figure 4 shows the values of 1/Δ*H*_HBx_ plotted with respect to the corresponding AFM peak heights, revealing a weak relationship between Δ*H*_HBx_ and the corresponding AFM IgG peak height; the reason for this weak dependence will be clarified in a future study. A rug plot of all of the data points for 1/Δ*H*_HBx_ along the *y*‐axis is displayed on the right‐hand side of the inset in Figure 4, revealing that the maximum density distribution of the values of Δ*H*_HBx_ appeared at ≈1 nm. Furthermore, when excluding the 40% of data from the top and bottom, the average magnitude of Δ*H*_HBx_ was ≈0.88 nm, with a standard deviation of 0.433 nm. According to the molecular mass of ≈17.8 kDa for a HBx protein, a single HBx protein can be estimated to have dimensions of only a few nanometers.^[^ ^30^ ^]^ Thus, we can reasonably assign our experimental observation of the average value of Δ*H*_HBx_ being ≈1 nm to the conformational change induced upon binding a single HBx protein to the binding site of an immobilized IgG antibody, forming an IgG–HBx complex.

Thanks to mature semiconductor manufacture technologies (allowing the fabrication of flat reactive surfaces and the definition of the miniature alignment mark for graphically superimposed alignment) and commercial AFM systems (with spatial analysis ability at the sub‐nanometer resolution), the ex situ AFM–molecule chip technique can be used for ex situ detection of antibody–antigen interactions at the single‐molecule level. To meet the technical requirements of practical disease diagnosis, further development of the ex situ AFM–molecule chip technique will be required. For instance, from the viewpoint of analysis, a fast AFM system equipped with an automatic chip‐loading system could be used to greatly shorten the operational time of each AFM analysis and simplify the technical procedure of chip loading into the AFM system. In addition, a procedure for AFM image analysis could be developed by adopting an artificial intelligence technique to 1) accelerate graphical data processing of the graphically superimposed alignment of the ex situ AFM mapping images recorded at the various stages and 2) identify the binding interaction between the biological target and its probe. Furthermore, other AFM analysis modules and other scanning probe microscopy techniques could also be used to simultaneously record various other surface characteristics of the chip surface at the single‐molecule level, not only limiting the system to topographical information, suggesting versatile applications of the ex situ AFM–molecule chip technique in the chemistry–biology world.

## 3 Conclusions

In summary, we have developed an ex situ AFM–molecule chip system with graphically superimposed alignment that enables ex situ AFM detection of antibody–antigen binding on a semiconductor chip surface at the single‐molecule level. A designed pattern on the chip surface acts as a mark for precise alignment of AFM mapping images recorded at various stages of the detection procedure, thereby allowing monitoring of the evolution of the topographical characteristics in the AFM analysis area. Accordingly, through statistical analysis, we could identify individual units of IgG antibodies covalently immobilized on an aldehyde‐terminated chip surface, with an AFM height of 5.416 nm, suggesting a predominantly head‐on orientation. Information on the changes in AFM‐measured heights of individual and aggregated IgG antibodies, arising from conformational changes upon the formation of IgG–HBx complexes, could also be determined statistically. Our system for ex situ AFM analysis appears to allow the detection of individual antibody–antigen binding events in a more practical, flexible, and versatile manner than previously developed in situ methods, while also potentially allowing other explorations of the biological interactions of antigen–antibody systems from the single‐molecule perspective.

## 4 Experimental Section

4.1

4.1.1

##### Chip Fabrication

An SOI wafer was adopted to simplify the semiconductor process flow for fabricating the semiconductor chip with the graphically superimposed alignment mark. After etching to remove the top‐layer silicon area excluding the area for the alignment mark, a silicon dioxide (SiO_2_) layer (thickness: 150 nm) was exposed, acting as a chemical reactive surface for the chemical/biological treatment processes.

##### Surface Chemical Modification

To clean its surface, the chip was immersed sequentially in acetone and EtOH, each for 10 min of ultrasonic treatment. The chip surface was then treated with oxygen plasma (power: 18 W; pressure: 550–650 mtorr; 3 min) to remove any organic contaminants and enhance the hydrophilicity of the SiO_2_ surface. Next, the chip was immersed in an EtOH solution containing 2% (3‐aminopropyl)triethoxysilane (APTES) for 30 min for surface silanization. The chip was rinsed with EtOH several times to remove any APTES residue from its surface, followed by drying for 10 min on a hot plate at 120 °C. Subsequently, to introduce aldehyde functional groups onto the surface, the APTES‐modified chip was immersed in 10 mM bis‐tris propane (BTP) buffer solution (pH 7) containing 2.5% glutaraldehyde (GA) for 1 h at room temperature, followed by several washes with BTP buffer. The chemical modification process is described elsewhere.^[^ ^31^ ^]^

##### Antibody Immobilization

The hepatitis B virus X protein (HBx; no. GTX17526‐pro) and the mouse IgG monoclonal antibody of the HBx protein (anti‐HBx; no. GTX22741) were purchased from GeneTex. The GA‐terminated chip was placed in a standard laboratory container along with 10 mM BTP buffer containing 10 μg mL^−1^ anti‐HBx and left at 4 °C for 16 h. Subsequently, the chip was rinsed several times with 10 mM BTP buffer (pH 7) and then subjected to chemical blocking of the unreacted aldehyde units through exposure to 10 mM tris(hydroxymethyl)aminomethane hydrochloride (Tris‐HCl) buffer containing sodium cyanoborohydride (NaBH_3_CN). Finally, the antibody‐modified chip was dried with N_2_ gas and stored in a vacuum bag at 4 °C until required for further experiments.

##### Antigen Incubation

After AFM analysis of the antibody‐immobilized chip, incubation with the antigen was executed immediately by placing the chip in a standard laboratory container along with 10 mM BTP buffer (pH 7) containing 5 μg mL^−1^ HBx and left for 30 min at room temperature. After incubation, the chip was rinsed several times with BTP buffer and then dried with N_2_ gas. AFM analysis of the chip surface was conducted immediately.

##### AFM Analysis

For ex situ AFM analysis, a Bruker Dimension Icon atomic force microscope was operated in tapping mode to analyze the topographical characteristics of the semiconductor chip surface under ambient conditions. The AFM tip having a nanoscale tip radius of curvature was purchased from NANOSENSORS^TM^ (PPP‐NCSTR). NanoScope Analysis software was used for data analysis of the AFM topographical mappings.

##### Statistical Analysis

Independent experimental sets (*N* > 5) were used for analysis. Statistical information, including the average value and standard deviation (*σ*) of the analysis data, is provided in the main text. The sample size of the data for each statistical analysis is described in the relevant figure captions.

## Conflict of Interest

The authors declare no conflict of interest.

## Authors Contributions

M.P.L. proposed the concept of the single‐molecule ex situ AFM–molecule chip. M.P.L. designed and fabricated the semiconductor chips. M.P.L., Y.Y.W., and Y.S.Y. discussed the detailed experiments regarding the surface functionalization, antibody immobilization, and antigen incubation. Y.Y.W. conducted the chemical and biological experiments. M.P.L. analyzed and characterized all of the experimental results from the AFM measurements. M.P.L., Y.Y.W., and Y.S.Y. discussed the experimental results. M.P.L. drafted the manuscript.

## Supporting information

Supplementary Material
