# Supplementary material for: Single‐Molecule Ex Situ Atomic Force Microscopy Allows Detection of Individual Antibody–Antigen Interactions on a Semiconductor Chip Surface
Source: Adv Nanobiomed Res. 2020 Dec 18;1(2):2000035. doi: 10.1002/anbr.202000035 (PMC7744838; doi:10.1002/anbr.202000035)
Supplement: Supplementary file 1 — Supplementary Material [file ANBR-1-0-s001.pdf]

Supporting Information

Single-Molecule *Ex Situ* Atomic Force Microscopy Allows Detection of Individual Antibody–Antigen Interactions on a Semiconductor Chip Surface

Ming-Pei Lu, Ying-Ya Weng, and Yuh-Shyong Yang*

Dr. Ming-Pei Lu
Taiwan Semiconductor Research Institute, National Applied Research Laboratories, Hsinchu 30078, Taiwan
E-mail: mingpei.lu@gmail.com; mplu@narlabs.org.tw

Ying-Ya Weng
Department of Electrical and Computer Engineering, Biomedical Engineering,
National Chiao Tung University, Hsinchu, Taiwan

Prof. Yuh-Shyong Yang
Department of Biological Science and Technology
National Chiao Tung University, Hsinchu, Taiwan

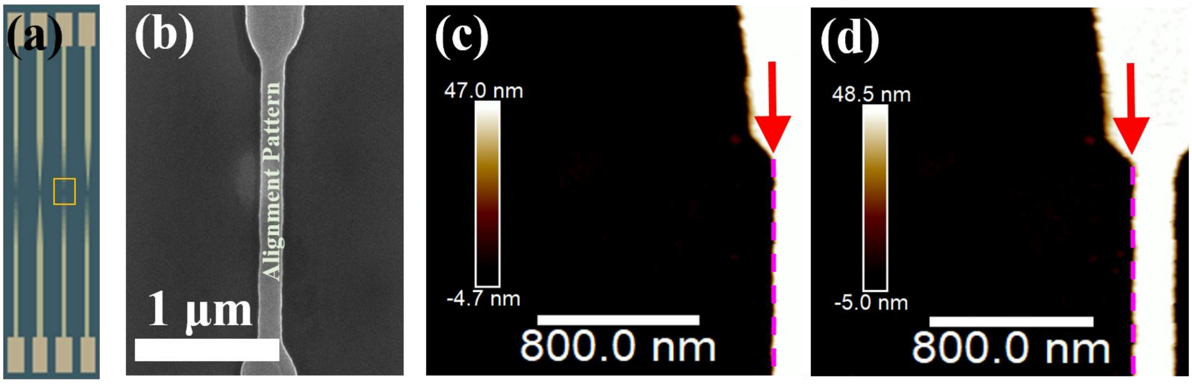

Figure S1. (a) Top-view optical microscopy (OM) image of a semiconductor chip designed with multiple analysis patterns distributed in parallel. The alignment pattern on the chip surface was readily identified by the OM system to facilitate movement of the AFM tip to the analysis area (located in the middle area of the pattern, highlighted by the yellow square). (b) Image of a typical analysis area captured by a scanning electron microscope, revealing a nanoscale designed pattern for graphically superimposed alignment. (c, d) AFM topographical mapping images recorded after (c) immobilization of anti-HBx IgG and (d) incubation with the HBx protein; the selected reference point on the alignment pattern is denoted by the red arrow.

In our experiments, the alignment pattern on the semiconductor chip surface featured two critical parts serving different purposes. The first was a micro-sized designed pattern that could be identified readily by the OM system to guide the movement of the AFM tip to the analysis area, as displayed in Figure S1a. The second part was a nanoscale-structured pattern (as displayed in Figure S1b) located within the analysis area, acting as a graphically superimposed mark for precise alignment of the AFM mapping images recorded at each stage of the detection procedure. After completing the AFM measurements on the same analysis area, we could use the alignment pattern to align all of the AFM topographical mapping images recorded at each stage of the detection procedure. For instance, we could intentionally select a specific structured location on the alignment pattern as the reference point. In Figures S1c and S1d, the selected reference point is denoted by a red arrow. The two images were first superimposed to align the selected reference points in all images. Next, the edges of the alignment pattern, denoted by the purple dashed lines in Figures S1c and S1d, were aligned to precisely correct the rotation angle of all of the AFM mapping images. Variations of the rotation angle of the analysis chip relative to the AFM scanning stage system could not be avoided precisely after the chip had been reloaded onto the AFM stage after completion of the chemical/biological process. Using the concept of graphically superimposed alignment, the *ex situ* AFM topographical mappings from each stage of the detection procedure could be precisely aligned, thereby allowing topographical differences at the same surface location to be identified between the various stages of the detection procedure. Notably, we performed the graphically superimposed alignment of the *ex situ* AFM topographical mappings manually, using commercial graphical software. We believe that the development of artificial intelligence techniques would accelerate the graphical data processing of the *ex situ* AFM topographical mappings. In addition, the design of the alignment pattern is flexible; it could be varied according to the requirements of a specific analysis and the technical conditions of the SPM modules.
